# Genetic Knock-Down of HDAC7 Does Not Ameliorate Disease Pathogenesis in the R6/2 Mouse Model of Huntington's Disease

**DOI:** 10.1371/journal.pone.0005747

**Published:** 2009-06-01

**Authors:** Caroline L. Benn, Rachel Butler, Lydia Mariner, Jude Nixon, Hilary Moffitt, Michal Mielcarek, Ben Woodman, Gillian P. Bates

**Affiliations:** 1 Department of Medical and Molecular Genetics, King's College London School of Medicine, King's College London, London, United Kingdom; 2 Pfizer Regenerative Medicine, UCB Granta Park, Great Abington, Cambridge, United Kingdom; 3 Nuffield Laboratory of Ophthalmology, University of Oxford, The John Radcliffe Hospital, Oxford, United Kingdom; Sapienza University of Rome, Italy

## Abstract

Huntington's disease (HD) is an inherited, progressive neurological disorder caused by a CAG/polyglutamine repeat expansion, for which there is no effective disease modifying therapy. In recent years, transcriptional dysregulation has emerged as a pathogenic process that appears early in disease progression. Administration of histone deacetylase (HDAC) inhibitors such as suberoylanilide hydroxamic acid (SAHA) have consistently shown therapeutic potential in models of HD, at least partly through increasing the association of acetylated histones with down-regulated genes and by correcting mRNA abnormalities. The HDAC enzyme through which SAHA mediates its beneficial effects in the R6/2 mouse model of HD is not known. Therefore, we have embarked on a series of genetic studies to uncover the HDAC target that is relevant to therapeutic development for HD. HDAC7 is of interest in this context because SAHA has been shown to decrease *HDAC7* expression in cell culture systems in addition to inhibiting enzyme activity. After confirming that expression levels of *Hdac7* are decreased in the brains of wild type and R6/2 mice after SAHA administration, we performed a genetic cross to determine whether genetic reduction of *Hdac7* would alleviate phenotypes in the R6/2 mice. We found no improvement in a number of physiological or behavioral phenotypes. Similarly, the dysregulated expression levels of a number of genes of interest were not improved suggesting that reduction in *Hdac7* does not alleviate the R6/2 HD-related transcriptional dysregulation. Therefore, we conclude that the beneficial effects of HDAC inhibitors are not predominantly mediated through the inhibition of HDAC7.

## Introduction

Huntington's disease (HD) is an autosomal dominant late-onset progressive neurodegenerative disorder with a mean age of onset of 40 years. Symptoms include psychiatric disturbances, motor disorders, cognitive decline and weight loss, disease duration is 15–20 years and there are no effective disease modifying treatments [1]. The HD mutation is an expanded CAG trinucleotide repeat in the *HD* gene that is translated into a polyglutamine (polyQ) repeat in the huntingtin (Htt) protein [2]. Neuropathologically, the disease is characterized by neuronal cell loss in the striatum, cortex and other brain regions and the deposition of nuclear and cytoplasmic polyQ aggregates [3], [4]. The R6/2 mouse model expresses exon 1 of the human HD gene with more than 150 CAG repeats [5], [6]. The R6/2 phenotype has an early onset and rapid and reproducible phenotype progression that recapitulates many features of the human disease. Motor and cognitive abnormalities can be detected before 6 weeks of age [7], [8], and mice are rarely kept beyond 15 weeks. PolyQ aggregates are clearly apparent in some brain regions from 3 to 4 weeks of age and striatal cell loss has been documented at later stages [9]. This suggests that the mouse phenotype is predominantly caused by neuronal dysfunction.

Transcriptional dysregulation occurs early in the molecular pathology of HD and has been recapitulated across multiple HD model systems (reviewed in [10]). RNA Affymetrix expression profiles of brain regions and muscle from both the R6/2 transgenic mouse and knock-in mouse models of HD show high correlation to expression profiles from HD post-mortem tissue [11]–[13]. The molecular mechanisms that underlie these selective transcriptional disturbances are unknown and remain the subject of investigation. The control of eukaryotic gene expression in part depends on the modification of histone proteins associated with specific genes with the acetylation and deacetylation of histones playing a critical role in gene expression [14]–[16]. Studies in numerous HD models have shown that mutant huntingtin expression leads to a change in histone acetyltransferase (HAT) activity and suggest that aberrant HAT activity may contribute to transcriptional dysregulation in HD [17]–[19]. Supporting this view, administration of histone deacetylase (HDAC) inhibitors such as suberoylanilide hydroxamic acid (SAHA) consistently shows therapeutic potential in HD models [20]–[28], at least partly through increasing the association of acetylated histones with down-regulated genes and correcting mRNA abnormalities [29].

There are three major classes of mammalian HDACs, based on their structural homology to the three *Saccharomyces cerevisiae* HDACs: rpd3 (class I), hda1 (class II) and sir2 (class III). Class I comprises HDAC1, -2, -3 and -8, class IIa HDAC4, -5, -7 and -9, class IIb HDAC6 and -10 and HDAC11 (class IV) shows homology to both rpd3 and hda1 [30]. Pan-HDAC inhibitors such as SAHA target the zinc-dependent HDACs 1–11 and not the NAD+ dependent class III HDACs (the seven sirtuins, SIRT1-7) [31]. In order to gain insight into which HDACs must be inhibited in order to alleviate HD-related phenotypes, genetic approaches have been used to complement pharmacology in *Drosophila melanogaster* and *Caenorhabditis elegans* HD models [19], [32]. However, as HDACs show differential levels of evolutionary conservation [33], the extent to which these studies will inform drug development in man has yet to be demonstrated.

The molecular mechanisms by which SAHA exerts its beneficial and toxic effects are currently not clear, nor whether the beneficial and toxic effects can be dissociated. We therefore need to systematically interrogate known targets of SAHA genetically in order to identify the HDAC(s) that present therapeutic targets relevant to HD. As a consequence, we have embarked on a serried of genetic crosses between the R6/2 mouse and specific HDAC knock-out mouse lines. It has previously been shown that in addition to enzyme inhibition, SAHA treatment selectively suppresses expression of *HDAC7 in vitro* [34], thus representing a potential molecular avenue by which pan-HDAC inhibition exerts a beneficial effect. We confirmed that chronic administration of SAHA decreases *Hdac7* mRNA expression levels in mouse brain irrespective of the *HD* genotype. *Hdac7* knockout mice were previously generated through a targeted inactivation of the endogenous murine *Hdac7* gene [35]. *Hdac7* null mice are embryonic lethal and die at E11.0. However, *Hdac7* heterozygote knockout mice were found to be viable and fertile, with no overt phenotype. We demonstrate that *Hdac7* mRNA and protein levels are reduced in *Hdac7*+/− heterozygous knock-out mice and that *Hdac7* expression is not altered by the presence of the R6/2 transgene. We performed a genetic cross between R6/2 mice and *Hdac7*+/− heterozygotes and found that genetic knock-down of *Hdac7* fails to confer any improvement to a number of physiological, behavioral and transcriptional phenotypes. We conclude that neither inhibition of HDAC7 nor its downregulation contributed significantly to the beneficial effects that we observed upon administration of SAHA.

## Results

### The expression of *Hdac7* is decreased in R6/2 brain in response to chronic treatment with SAHA

**Figure 1:**
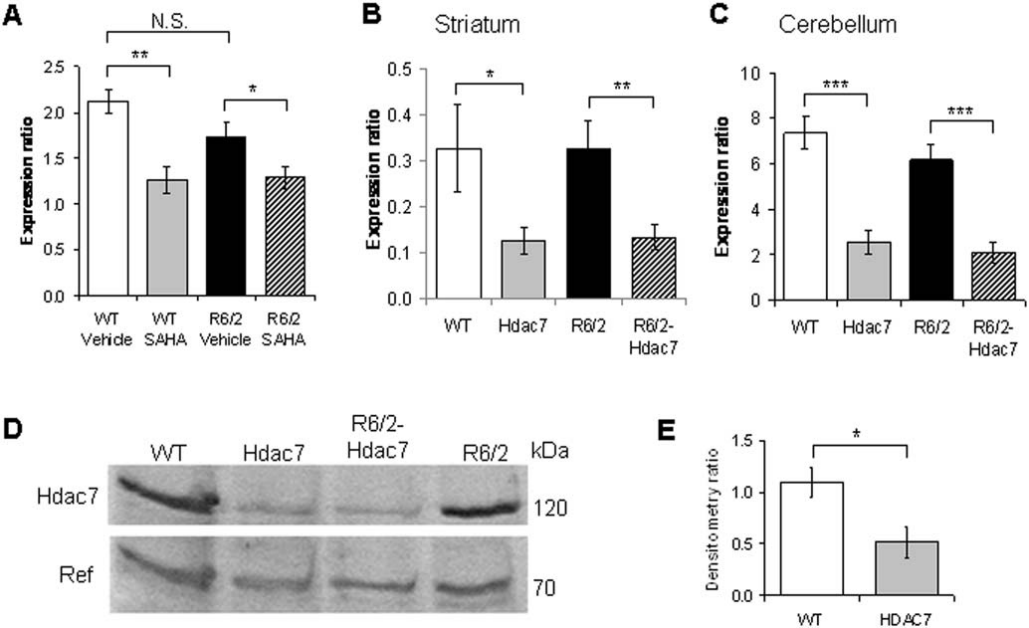
SAHA specifically down regulates *Hdac7* mRNA levels. *Hdac7* expression is decreased in *Hdac*7+/− mice and not altered by the presence of the R6/2 transgene. (A) *Hdac7* mRNA expression levels are shown as relative expression ratios to the geometric mean of the *Actb* and *Grin1* housekeeping genes in 13 week old mouse cerebellum from SAHA-treated WT (grey) and R6/2 (black) mice together with vehicle treated WT (white) and R6/2 (striped) mice (n>8 per genotype). Error bars are S.E.M. (B, C) *Hdac7* expression level is shown as a relative expression ratio to the geometric mean of three housekeeping genes in (B) striatum and (C) cerebellum of 14 week old wild-type (Wt, white), *Hdac7+/−* knockdown (Hdac7) (grey), R6/2 (black) and R6/2 mice with *Hdac7*+/− knockdown (R6/2-*Hdac7*, striped) mice (n>8 per genotype). Error bars are S.E.M. (D) Representative western immunoblot of 50 µg of cortical homogenate from 14 week old wild-type (WT), R6/2, *Hdac7*+/− and R6/2-*Hdac7*+/− mice. Blots were probed with an antibody that recognizes Hdac7 (120 kDa) and a non-specific band (70 kDa). (E) Quantification of Hdac7 protein expression levels in WT (white) and *Hdac7*+/− (grey) mice. Quantification was performed on blots containing four samples per genotype using the non-specific band for reference. Blots were additionally probed with an antibody to α-tubulin to confirm equal protein loading (data not shown). Error bars represent standard deviation from the mean (*n* = 4). * p<0.05, ** p<0.01, *** p<0.001.

SAHA has been described as a pan-HDAC enzyme inhibitor [36], [37]. In addition, it was recently shown that treatment of a number of cell lines with SAHA resulted in the specific down-regulation of *HDAC7* at the mRNA level [34]. To determine whether the expression of *Hdac7* might be similarly altered by the administration of SAHA *in vivo*, we established a quantitative real time PCR assay (RT-qPCR) for murine *Hdac7*. We had previously conducted an efficacy trial to assess the effects of the administration of SAHA, when complexed with hydroxypropyl-β-cyclodextrin at a concentration of 0.67 g/L in the drinking water, and observed a considerable improvement in RotaRod performance [23]. The WT and R6/2 mice that had been treated with SAHA or vehicle in this experiment had been sacrificed at 13 weeks of age and we still had access to cerebellar cDNA that had been prepared from the brains of these mice. We were able to show that SAHA significantly decreased the expression of *Hdac7* in the cerebellum of both wild type (WT) and R6/2 mice (Figure 1A). Therefore, SAHA has a similar effect on the expression of *Hdac7 in vivo* as has been described in cell culture and this could contribute to the beneficial effects of SAHA administration.

### *Hdac7* expression level is unaffected by the expression of the R6/2 transgene

We found heterozygous *Hdac7* knockout mice to be viable and fertile, with no overt phenotype. Therefore, prior to interpreting the results of a genetic cross to determine whether heterozygous knock-down of *Hdac7* has a beneficial effect on R6/2 HD-related phenotypes, it was important to show that (1) *Hdac7* expression is reduced in *Hdac7*+/− knock-out mice: that *Hdac7* is not auto-regulated to wild type levels as is the case for *Hdac1* [38] and (2) that the presence of the R6/2 transgene does not alter *Hdac7* expression levels. RT-qPCR was performed on cDNA prepared from the striatum and cerebellum of 15 week old wild type (WT), *Hdac7* heterozygote (*Hdac7*+/−), R6/2 transgenic (R6/2) and R6/2 mice heterozygote for *Hdac7* (R6/2-*Hdac7*+/−) mice. We found that *Hdac7* expression levels were significantly decreased in the striatum and cerebellum of *Hdac7*+/− heterozygote mice, irrespective of the presence of the R6/2 transgene (Figure 1B and C). Furthermore, we saw no difference in *Hdac7* expression levels between WT and R6/2 mice in either brain region. Western blotting was performed on whole cell lysates from 15 week cortices. We found HDAC7 protein levels to be in agreement with the RNA expression profiling (Figure 1D and E).

### *Hdac7* is expressed in neuronal populations in the mouse brain

**Figure 2:**
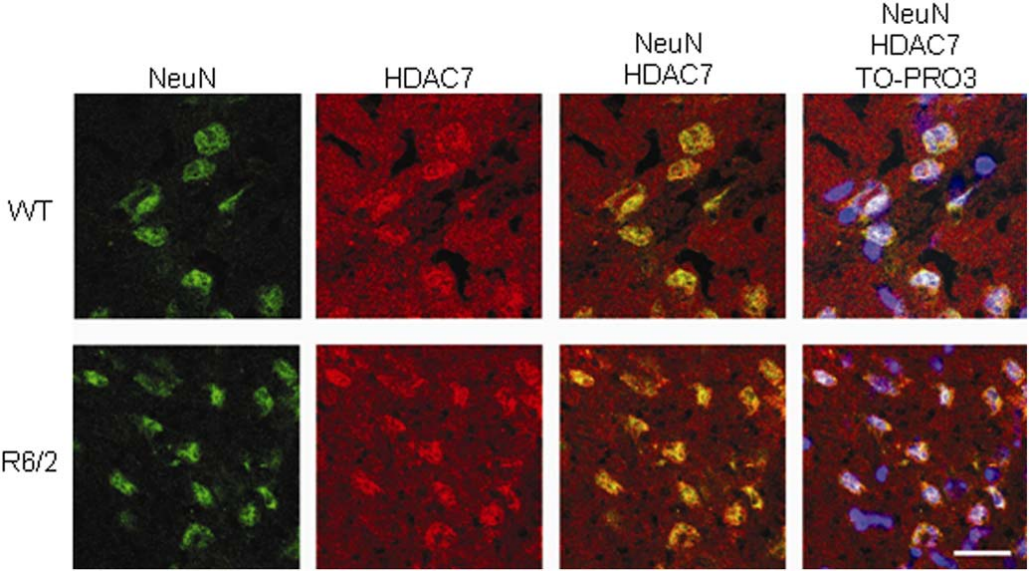
HDAC7 is present in the nucleus and cytoplasm of neuronal cells in the mouse striatum. Representative confocal microscopy images of coronal sections of brains from wild-type (WT) and R6/2 transgenic mice at 14 weeks of age immuoprobed with antibodies to NeuN to identify neurons and with anti-HDAC7. Nuclei were visualized using TO-PRO-3 (blue). Scale bar 20 µm.

An important feature of the class IIa HDACs is their ability to shuttle between the nucleus and the cytoplasm. Precise regulation of the subcellular distribution of class IIa HDACs is intimately linked to the control of their activity and plays a pivotal role in cellular processes and organ development [39]. Previous observations have highlighted a differential subcellular localization of HDAC7 in different cell lines and body tissues, suggesting that the control of subcellular localization and function of HDAC7 differs with respect to the cell type. To explore the expression pattern of Hdac7 in the mouse brain we performed immunohistochemistry to coronal sections of WT and R6/2 brains from animals at 14 weeks of age. HDAC7 is present in both the nucleus and cytoplasm in the striatum (Figure 2), cortex and cerebellum (data not shown) of both WT and R6/2 brains. It is present in all neurons, but interestingly appears to be absent from the nuclei of at least some non-neuronal cell populations.

### Genetic reduction of *Hdac7* does not modify the R6/2 phenotype

We have previously established a set of quantitative tests with which to monitor progressive behavioral phenotypes in R6/2 mice [40]–[42]. To generate mice for this analysis, male R6/2 mice were bred with female *Hdac*7 heterozygote knock-out mice (*Hdac*7+/−) to produce at least 10 female mice from each genotype (WT, n = 10; *Hdac7*+/−, n = 13; R6/2, n = 14; R6/2-*Hdac7*+/−, n = 12), which were born over a period of 5 days. The CAG repeat size was well matched between the R6/2 and R6/2-*Hdac*7+/− groups (P = 0.102) (Table 1). Weight gain, RotaRod performance, grip strength and exploratory activity were monitored from 4 to 15 weeks of age, and in each case, a specific test was performed on the same day and at the same time during the weeks in which measurements were taken.

**Table 1:** Details of mice used in the study.

Group	Sex	No. of mice	CAG Repeat Size
**Wild type**	Female	10	
***Hdac7*** **+/−**	Female	13	
**R6/2**	Female	14	202 (SD 3.64)
***Hdac7*** **+/−, R6/2**	Female	12	203 (SD 5.22)

**Figure 3:**
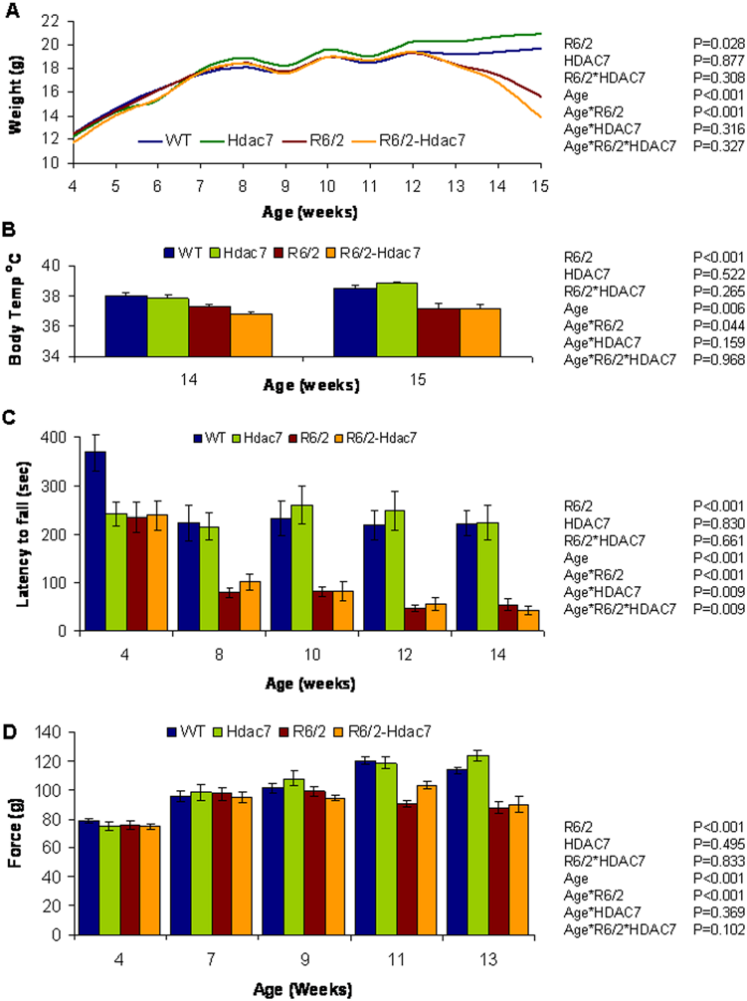
HDAC7 genetic reduction does not alter R6/2 physiological or behavioural phenotypes. The R6/2 phenotypes (A) weight loss, (B) hypothermia, (C) impaired RotaRod performance and (D) reduced grip strength are not ameliorated by genetic knock-down of *Hdac7* expression. Error bars represent S.E.M. Shown to the right of each graph are the P values arising from GLM repeated measures ANOVA analysis of the data.

Mice were weighed weekly from 4 to 15 weeks of age (Figure 3A). As expected, R6/2 mice weighed less overall than WT mice [F_(1,45)_ = 5.18, P = 0.028] and gained weight at a slower rate [F_(4,495)_ = 18.546, P<0.001] (Figure 3A). There was no difference in the overall weight [F_(1,45)_ = 0.024, P = 0.877] or in weight gain [F_(4,495)_ = 1.19, P = 0.316] between WT and *Hdac*7+/− mice. Genetic reduction of *Hdac*7 had no effect on R6/2 weight [F_(1,45)_ = 1.064, P = 0.308] and did not attenuate the rate of R6/2 weight loss [F_(4,495)_ = 1.168, P = 0.327]. Therefore, genetic reduction of *Hdac*7 does not improve the weight loss phenotype in the R6/2 mice.

The body temperature of the mice was recorded at 14 and 15 weeks of age by rectal probe (Figure 3B). R6/2 mice were found to be hypothermic [F_(1,45)_ = 69.781, P<0.001], which worsened over the course of the week between measurements [F_(1,45)_ = 4.315, P = 0.044]. There was no overall change in body temperature in the *Hdac*7+/− mice [F_(1,45)_ = 0.416, P = 0.552], nor with time [F_(1,45)_ = 2.05, P = 0.159]. There was no effect of the *Hdac*7+/− genotype on R6/2 body temperature [F_(1,45)_ = 1.276, P = 0.265], nor the progression of the R6/2 hypothermia [F_(1.45)_ = 0.002, P = 0.968]. Therefore, genetic reduction of *Hdac*7 does not improve the hypothermia that occurs in symptomatic R6/2 mice.

RotaRod performance is a sensitive indicator of balance and motor coordination, which has been reliably shown to decline in R6/2 mice [40]. Using this test, we found that R6/2 and R6/2-*Hdac*7+/− mice performed similarly with age and that the performance of *Hdac*7+/− and WT mice was equivalent (Figure 3C). Consistent with previous results, the overall RotaRod performance of R6/2 was impaired as compared to WT [F_(1 ,45)_ = 40.634, P<0.001] and deteriorated with age [F_(2 ,900)_ = 9.119, P<0.001]. Overall, the performance of *Hdac*7+/− mice did not differ from WT [F_(1,45)_ = 0.047, P = 0.83] but did change with age [F_(2,900)_ = 4.427, P = 0.009] however, this is most likely the result of the exceptionally good, but atypical, performance of the WT mice at four weeks. There was no overall effect of *Hdac*7 knock-down on the RotaRod performance of the R6/2 mice [F_(1 ,45)_ = 0.194, P = 0.661] and examination of the data (Figure 3C) indicates that the statistically significant interaction between the genotypes over the course of the experiment [F_(2,900)_ = 4.516, P = 0.009] is, once again, due to the performance of the WT mice at 4 weeks of age and not a reflection of an alteration in the R6/2 phenotype.

Forelimb grip strength was assessed at 4, 7, 9, 11 and 13 weeks of age (Figure 3D). Consistent with previous data, R6/2 mice performed significantly worse than WT [F_(1,45)_ = 16.588, P<0.001] and performance deteriorated with age [F_(3,360)_ = 13.248, P<0.001]. The grip strength of *Hdac*7+/− mice was comparable to WT mice, both overall [F_(1, 45)_ = 0.474, P = 0.495] and over the course of the experiment [F_(3,360)_ = 1.055, P = 0.369]. Genetic reduction of *Hdac*7 did not improve R6/2 grip strength overall [F_(1,45)_ = 0.45, P = 0.833] or with age [F_(3,360)_ = 2.131, P = 0.102].

Exploratory activity was assessed fortnightly from 5 to 13 weeks of age as described previously [41] and analyzed by repeated measures general linear model (GLM) ANOVA. Mice were assessed for a period of 60 min for total activity, mobility and rearing and the P-values obtained through the analyses are displayed in Table 2 for each parameter. Mice of all genotypes exhibit most activity during the first 15 minutes of the assessment period [41]. R6/2 mice show an overall hypoactivity relative to WT mice from 11 weeks (Table 2, R6/2) although the pattern of activity over the course of the 60 min period was significantly different between R6/2 and WT mice (R6/2*time) by five weeks of age. *Hdac*7+/− mice were indistinguishable from WT mice, both in overall activity (HDAC7) and in the 60 minute pattern of activity (HDAC7*time). There was no overall improvement in R6/2 hypoactivity through genetic reduction of *Hdac*7 (R6/2*HDAC7), nor was the R6/2 pattern of hypoactivity changed (R6/2*HDAC7*time). In summary, *Hdac*7 genetic reduction does not improve hypoactivity in the R6/2 mice.

**Table 2:** Statistical analysis of exploratory activity.

Factor	Week	Activity	Mobility	Rearing
Time	5	<0.001	<0.001	<0.001
	7	<0.001	<0.001	<0.001
	9	<0.001	<0.001	0.269
	11	<0.001	<0.001	0.667
	13	<0.001	<0.001	<0.001
R6/2	5	0.374	0.260	0.068
	7	0.990	0.632	0.772
	9	0.315	0.045	0.536
	11	0.012	0.001	0.035
	13	<0.001	<0.001	<0.001
Time*R6/2	5	0.224	0.357	0.448
	7	0.425	0.674	0.799
	9	0.096	0.033	0.104
	11	<0.001	<0.001	0.167
	13	<0.001	<0.001	0.052
HDAC7	5	0.579	0.973	0.307
	7	0.212	0.085	0.557
	9	0.128	0.116	0.740
	11	0.857	0.574	0.621
	13	0.506	0.497	0.915
Time*HDAC7	5	0.715	0.688	0.292
	7	0.027	0.099	0.465
	9	0.603	0.084	0.363
	11	0.558	0.255	0.072
	13	0.557	0.563	0.406
R6/2*HDAC7	5	0.689	0.397	0.774
	7	0.945	0.717	0.383
	9	0.643	0.688	0.779
	11	0.795	0.328	0.549
	13	0.312	0.113	0.753
Time*R6/2*HDAC7	5	0.106	0.371	0.470
	7	0.668	0.586	0.621
	9	0.418	0.509	0.428
	11	0.198	0.174	0.477
	13	0.143	0.174	0.214

The numbers displayed in table 2 indicate the P-values for each of the parameters analysed (genotype of mice, age of mice and time in the activity cages), with significant P-values highlighted for ease of interpretation. Mice in activity cages are shown to behave differently with respect to measures of exploratory behaviour (as determined by measuring activity, mobility and rearing) over a period of one hour (Time). Wild-type and R6/2 mice exhibit reproducible differences in measures of exploratory behaviour with R6/2 mice developing a progressive hypoactivity that becomes significant at older ages as demonstrated by decreased activity and mobility in addition to decreased rearing behaviour (R6/2 genotype and R6/2 genotype*time). Hdac7 reduction does not influence the behaviour of mice for any parameter assessed (Hdac 7 genotype and Hdac7*time) and furthermore, there appears no interaction between Hdac7 and R6/2 genotypes (R6/2*Hdac7 and R6/2*Hdac7*time).

### *Hdac7* genetic reduction does not ameliorate the dysregulated expression of genes of interest in R6/2 mouse brains

**Figure 4:**
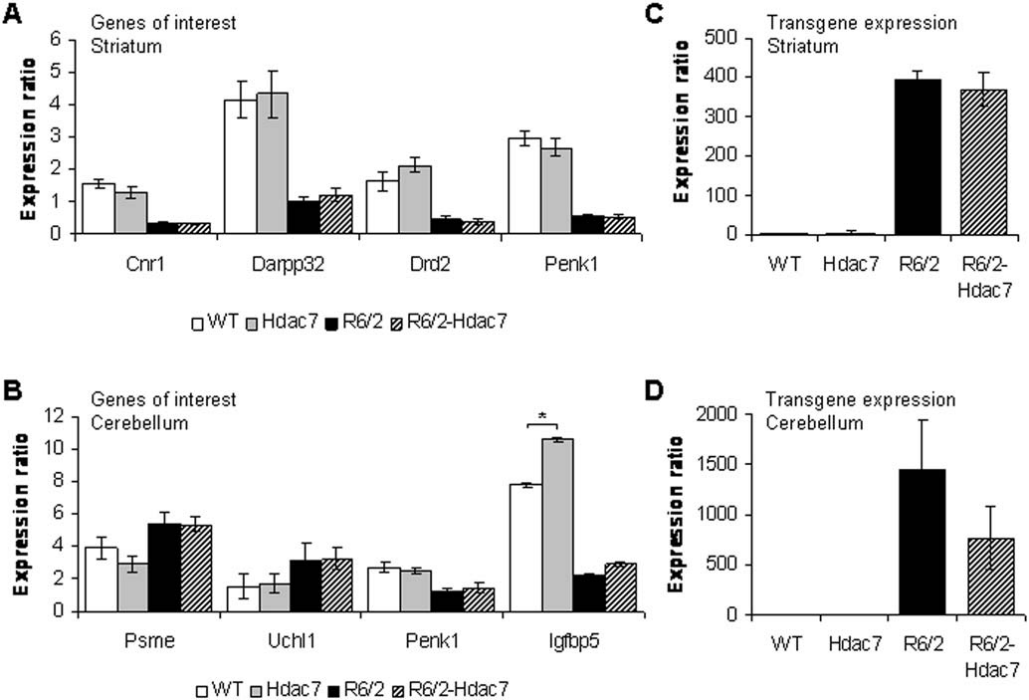
Comparable changes in striatal and cerebellar gene expression changes between R6/2 and R6/2-HDAC7 mice. Relative expression ratios to the geometric mean of three housekeeping genes [44] for transcripts in striatum (A) and cerebellum (B) of 15 week old WT (white), *Hdac7*+/− (grey), R6/2 (black) and R6/2-*Hdac7* (striped) mice. Error bars correspond to S.E.M. (n>8). * P<0.05. *Hdac7* genotype does not modulate R6/2 transgene expression level in striatum (C) or cerebellum (D) of 15 week old mice R6/2 (black) and R6/2-*Hdac7*+/− (striped). Error bars correspond to S.E.M. (n>8). *Cnr1*, cannabinoid receptor 1; *Darpp32*, Dopamine and cAMP regulated neuronal phosphoprotein; *Drd2*, dopamine D2 receptor; *Penk1*, proenkephalin; *Psme1*, proteasome activator subunit 1 (PA28 alpha) Reg alpha; *Uchl1*, ubiquitin C-terminal hydrolase L1; *Igfbp5*, insulin-like growth factor binding protein 5.

As HDAC inhibitors have been shown to ameliorate the dysregulation of gene expression in HD models systems [28], [29], [43], we used RT-qPCR to measure the level of expression of a set of genes of interest in the striatum and cerebellum of WT, R6/2, *Hdac7*+/− and R6/2-*Hdac7*+/− mice aged 15 weeks. These included striatal genes that are consistently down-regulated in mouse models of HD and in HD patient brains and cerebellar genes that have consistently altered expression patterns in both R6/2 and the *Hdh*Q150 knock-in mouse model of HD [13], [42], [44]. We found that *Hdac7* reduction did not ameliorate the transgene-mediated transcriptional dysregulation in the striatum (Figure 4A) or the cerebellum (Figure 4B) of 14 week old mice. However, the expression of *Igfbp5* was increased with HDAC7 reduction in the cerebellum of WT (P = 0.047) but not R6/2 mice (P = 0.690) (Figure 4B). Concomitantly, we found no difference in R6/2 transgene expression in the striatum (P = 0.620) (Figure 4C) and the cerebellum (P = 0.240) (Figure 4D) of R6/2 and R6/2-*Hdac7*+/− mice.

## Discussion

We have previously shown that chronic administration of the HDAC inhibitor SAHA to R6/2 mice significantly improves some motor and neuropathological phenotypes [23]. However, the therapeutic index of SAHA in mice is very narrow, and therapeutic doses show considerable toxicity resulting in weight loss in both WT and R6/2 mice. SAHA has been reported to inhibit the eleven Zn^2+^ dependent HDAC enzymes [37] and therefore dissection of the mechanism through which SAHA exerts its beneficial effects is complex. In order to identify the HDAC enzyme(s) that are therapeutic targets for HD and in an attempt to separate the beneficial effects of SAHA from its toxicity, we have embarked on a series of genetic crosses to mice that have been genetically engineered to knock-out specific HDAC enzymes. HDAC7 is of additional interest because SAHA has been shown to decrease the level of expression of *HDAC7* in a number of cell lines [34]. In this report we showed that these results extend to an *in vivo* system and that, after chronic administration, SAHA down-regulates *Hdac7* mRNA levels in the brains of WT and R6/2 mice.

As nullizygosity for *Hdac7* is embryonic lethal [35], it is not possible to investigate the effects of knocking-out the *Hdac7* gene on the R6/2 phenotype. However, reduced levels of Hdac7 might be more akin to the effects to the pharmacological inhibition of this enzyme. We therefore established whether an investigation of the effects of knocking-down *Hdac7* levels using *Hdac7*+/− knock-out mice would be feasible. This was necessary as it has been shown that the effects of the genetic knock-down of *Hdac1* cannot be studied because *Hdac1* mRNA and protein levels autoregulate to that of WT mice in *Hdac1*+/− mice [38] and furthermore, nullizygosity for *Hdac1* is embryonic lethal [45]. We thus confirmed that *Hdac*7 expression is reduced in both the striatum and cerebellum of *Hdac7*+/− mice and that the presence of the R6/2 transgene does not alter *Hdac7* expression levels in either *Hdac7*+/+ or *Hdac7*+/− mice at the mRNA or protein levels. We went on to show that genetic reduction of *Hdac7* levels did not impact on the body weight, body temperature, RotaRod performance, grip strength or exploratory activity of R6/2 mice. Similarly, decreased *Hdac7* expression did not ameliorate the HD-related dysregulated expression levels of a number of specific genes of interest.

Very little is currently known about the function of HDAC7 in brain. *Hdac7* has previously been shown to be expressed throughout the rat brain by *in situ* hybridization [46]. Crucially, we established that Hdac7 is present in neurons, but were surprised to find that it could not be detected by immunohistochemistry in at least a proportion of non-neuronal brain cells. HDAC7 together with HDACs 4, -5 and –9 comprise the class IIa HDACs, which share a high degree of homology at their C-terminal catalytic domain [47]. The class IIa HDACs shuttle between the nucleus and cytoplasm and precise regulation of their subcellular distribution plays a pivotal role in modulating their function, via post-translational modifications such as phosphorylation, and the formation of nucleocytoplasmic shuttling complexes [31], [47]–[60]. Of the class IIa HDACs, HDAC7 is the most divergent [47], [61]. HDAC7 has been shown to associate with transcription co-repressors and factors such as CtBP, MEF2, HP1α, SMRT, N-CoR, mSin3A, and HIF1α [16], [31], [39], [47], [49], [57]. These interactions are consistent with the role of HDAC7 in regulating gene expression either as a co-activator or a co-repressor. *In vitro* studies with recombinant HDAC7 protein have suggested that histones may be substrates of HDAC7 deacetylase activity [62]. However, it has been shown that modulating HDAC7 levels *in vitro* by siRNA knockdown or overexpression is associated with growth arrest without detectable changes in histone acetylation or p21 gene expression [34]. This would be consistent with HDACs having many protein substrates, in addition to histones, involved in the regulation of gene expression, cell proliferation, and cell death and thus HDACs can be considered to be “lysine deactelylases”. Although the functions of HDAC7 in brain are unknown and remain to be elucidated, our genetic studies lead us to conclude that inhibition of *Hdac*7 is not a major mediator of the beneficial effects that we obtained upon administration of SAHA to R6/2 mice and HDAC7 should not be prioritized as a therapeutic target for HD.

## Materials and Methods

### Mouse maintenance and breeding

Hemizygous R6/2 mice were bred and reared in our colony by backcrossing R6/2 males to (CBA x C57BL/6) F1 females (B6CBAF1/OlaHsd, Harlan Olac). Mice nullizygous for the *Hdac*7 gene are embryonic lethal but heterozygotes are viable and fertile. On arrival at our facility, the *Hdac7*+/− mice were on a SvEv129/C57Bl6 mixed background and once at King's College London were backcrossed to (C57Bl/6×CBA) F1 females seven times before breeding with R6/2 males. All animals had unlimited access to water and breeding chow (Special Diet Services, Witham, UK), and housing conditions and environmental enrichment were as previously described [40]. In the case of mice arising from the R6/2×*Hdac*7+/− cross, all cages contained at least one mouse from each genotype and mice were additionally given mash consisting of powdered chow mixed with water from 12 weeks of age. Mice were subject to a 12 h light∶12 h dark cycle. All experimental procedures were performed in accordance with Home Office regulations.

### Genotyping and CAG repeat sizing

R6/2 mice were identified by PCR of tail-tip DNA. For R6/2, a 10 µl reaction contained 100 ng DNA, 1× Thermo-Start master mix (Thermo Scientific), 1 µl DMSO, 10 ng/µl forward primer 33727 [5′-CGCAGGCTAGGGCTGTCAATCATGCT-3′], and 10 ng/µl reverse primer 32252 [5′-TCATCAGCTTTTCCAGGGTCGCCAT-3′]. Cycling conditions were: 15 min @ 94°C, 35× (30 s @ 94°C; 30 s @ 60°C, 60 s @72°C) 10 min @ 72°C. The amplified R6/2 transgene product was 272 bp. Amplification of the CAG repeat from R6/2 mouse DNA was performed with a FAM labelled forward primer (GAGTCCCTCAAGTCCTTCCAGCA) and reverse primer (GCCCAAACTCACGGTCGGT) in 10 µl reactions containing: 0.2 mM dNTPs; 10% DMSO; AM buffer (67 mM TrisHCL pH 8.8; 16.6 mM (NH_4_)S0_4_; 2 mM MgCl_2_; 0.17 mg/ml BSA) and 0.5 U AmpliTaq DNA polymerase (Applied Biosystems). Cycling conditions were: 90 s @ 94°C, 24× (30 s @ 94°C; 30 s @ 65°C; 90 s @ 72°C) 10 min @ 72°C. All instruments and materials were obtained from Applied Biosystems unless indicated. The FAM-tagged PCR product (1 µl) together with MegaBACE™ ET900 (Amersham Bioscience) internal size standard (0.04 µl) were denatured at 94°C, 5 min in 9 µl of HiDi-formamide and analysed using an ABI3730 sequencer. Data analysis was performed using plate manager application GeneMapper v5.2- 3730XL.

*Hdac7*+/− mice were genotyped by multiplex PCR as follows: a 25 µl reaction contained 2 µl tail-tip genomic DNA (100 ng/µl), 2.5 µl 2 mM dNTPs, 5 µl 5× Promega Buffer, 1.5 µl 25 mM MgCl_2_, 2.5 µl DMSO, 1 µl (of 10 µM stock) primer “geno-SA 3” sequence (5′–3′): GTTGCAGGGTCAGCAGCGCAGGCTCTG, 0.45 µl (of 10 µM stock) primer “geno-SA 5-2” Sequence (5′–3′): CCAGTGGACGAGCATTCTGGAGAAAGGC, 0.55 µl (of 10 µM stock) primer “lacZ 3-2” Sequence (5′–3′): GCCAGTTTGAGGGGACGACGACAGTATCG, 0.2 µl Promega *Taq* (5 U/µl), and 12. 5 µl ddH_2_O. Cycling conditions were as follows: 95°C, 5 min, 35× (95°C, 30 s; 60°C, 30 s; 72°C, 45 s) and 72°C, 5 min before holding at 4°C. Wild-type product size was 400 bp whereas Het mice were indicated by a band at 640 bp.

### Phenotype analysis

Mice were weighed weekly to the nearest 0.1 g. Motor coordination was assessed using an Ugo Basile 7650 accelerating RotaRod (Linton Instrumentation, UK), modified as previously described [40]. At 4 weeks of age, mice were tested on four consecutive days, with three trials per day. At 8, 10, 12 and 14 weeks of age, mice were tested on three consecutive days with three trials per day. Forelimb grip strength was measured once a week at 4, 7, 9, 11 and 13 weeks using a San Diego Instruments Grip Strength Meter (San Diego, CA, USA) as described [40]. Exploratory, spontaneous motor activity was recorded and assessed every two weeks at 5, 7, 9, 11 and 13 weeks of age for 60 min during the day using AM1053 activity cages, as described previously [41]. Briefly, activity (total number of beam breaks in the lower level), mobility (at least two consecutive beam breaks in the lower level) and rearing (number of rearing beam breaks) were measured. The data were collected and analyzed as described previously.

### RNA extraction and real-time PCR expression analysis

RNA extraction and reverse transcription of 4 µg of total cerebellar RNA and 1 µg total striatal RNA was performed as previously described [44]. The RT reaction was diluted 10-fold in nuclease free water (Sigma) and 5 µl was used in a 25 µl reaction containing Precision MasterMix (PrimerDesign), 400 nM primers and 300 nM probe using the Opticon 2 real-time PCR machine (MJ Research). Estimation of mRNA copy number was determined in duplicate for each RNA sample by comparison to the geometric mean of two or three endogenous housekeeping genes as described [44]. Primer and probe sequences are available in Supplementary Table S1.

### Antibodies and western blotting

Mouse brains were rapidly frozen in liquid nitrogen and stored at −80°C. Cortices were homogenized in ice cold buffer containing 0.32 mM sucrose, 10 mM Tris-HCl, 50 mM KCl, 1 mM EDTA, 0.1% Triton X-100, protein inhibitor cocktail (Roche) with the final pH adjusted to 7.8, followed by sonication for 6×10 s on ice (amplitude 30) (Vibracell Sonicator) and centrifugation at 2500×*g* for 15 min at 4°C. Protein concentration was determined using the BCA assay kit (Perbio). 50 µg protein lysates was fractionated on 8% SDS-PAGE gels and transferred into the Protran nitrocellulose membrane (Schleicher and Schuell) by submerged transfer apparatus (Bio-Rad) in (25 mM Tris, 192 mM glycine, 20%, v/v, methanol). Membranes were blocked for 30 min. at RT in 5% non-fat dried milk in PBS-Tween-20, incubated with gentle agitation over night at 4°C with the Rabbit anti-human HDAC7 antibody raised against peptide 1-13aa (1∶250, CHDI Foundation) or alpha-Tubulin (1∶40000, Sigma) (in PBS-Tween-20 with 1% non-fat dried milk). For chemiluminescent detection, blots were washed three times in PBST (PBS, 0.1% Tween-20), probed with HRP-linked secondary antibodies (HRP conjugated anti-rabbit mouse or anti-antibody (1∶3000, Dako) (in PBS-Tween20 with 1% non-fat dried milk) for 1 h at RT and washed three times in PBST. Protein was detected by chemiluminescense (Femto WB detection system (Pierce) according to the manufacturer's instructions. The signals were quantified using a GS-800 calibrated densitometer (Bio-Rad).

### Immunohistochemistry and confocal microscopy

Whole brains were frozen in isopentane, stored at 80°C and 15 µm thick sections were cut using a cryostat (Bright Instruments Ltd.). Sections were fixed for 10 min in methanol at −20°C and washed twice in 0.1 M phosphate buffered saline pH 7.4 (PBS) for 15 min before blocking in PBS containing 2% bovine serum albumin (BSA) and 0.1% Triton-X for 15 min. Sections were incubation in primary antibodies in PBS with BSA overnight at 4°C, washed twice in PBS for 15 min, incubated in secondary fluorescent antibodies in PBS with BSA for 1 hr at room temperature and washed twice in PBS for 15 min. Primary antibodies were: HDAC7 (rabbit polyclonal Sigma H2662) (1∶50) and NeuN (mouse monoclonal Chemicon MAB377) (1∶200) and secondary antibodies were: Alex-555 donkey anti-rabbit (1∶1000) and Alexa 488 goat anti-mouse (1∶1000) respectively (Molecular Probes). Nuclei were visualized using TO-PRO-3 (Molecular Probes) (1∶1000). Slides were mounted in Mowial and antibody location was visualized using an LSM150 Meta confocal microscope (Zeiss).

### Statistical analysis

Statistic analysis was performed by Student's t-test (Excel or SPSS), one-way ANOVA, two-way ANOVA and repeated measures GLM ANOVA, with the Greenhouse–Geisser correction for non-sphericity using SPSS.

## Supporting Information

Table S1 Sequences of primers and Taqman probes used in real-time PCR assays. KEY: Cnr1 (Cannabinoid receptor 1); Darpp32 (Dopamine and cAMP regulated neuronal phosphoprotein, also known as Ppp1r1b, protein phosphatase 1, regulatory subunit 1B); Drd2 (Dopamine D2 receptor); Hdac (histone deacetylase); Htt (Human Huntington's disease gene, used to detect human exon 1 transgene); Igfbp5 (insulin-like growth factor binding protein 5 precursor); Pcp4 (Purkinje cell protein 4); Penk1 (Preproenkephalin); Uchl1 (ubiquitin carboxyl-terminal hydrolase L1).
(0.03 MB DOC)
