# Supplementary material for: Genetic Knock-Down of HDAC7 Does Not Ameliorate Disease Pathogenesis in the R6/2 Mouse Model of Huntington's Disease
Source: PLoS One. 2009 Jun 1;4(6):e5747. doi: 10.1371/journal.pone.0005747 (PMC2684627; doi:10.1371/journal.pone.0005747)
Supplement: Table S1 — Sequences of primers and Taqman probes used in real-time PCR assays. KEY: Cnr1 (Cannabinoid receptor 1); Darpp32 (Dopamine and cAMP regulated neuronal phosphoprotein, also known as Ppp1r1b, protein phosphatase 1, regulatory subunit 1B); Drd2 (Dopamine D2 receptor); Hdac (histone deacetylase); Htt (Human Huntington's disease gene, used to detect human exon 1 transgene); Igfbp5 (insulin-like growth factor binding protein 5 precursor); Pcp4 (Purkinje cell protein 4); Penk1 (Preproenkephalin); Uchl1 (ubiquitin carboxyl-terminal hydrolase L1). (0.03 MB DOC) [file pone.0005747.s001.doc]

**Table S1: Sequences of primers and Taqman probes used in real-time PCR assays**

| **GENE** | **FORWARD PRIMER** | **REVERSE PRIMER** | **PROBE (5’ 6-FAM; 3’ TAMRA)** |
| --- | --- | --- | --- |
| *Cnr1* | CACAAGCACGCCAATAACACA | ACAGTGCTCTTGATGCAGCTTTC | CCAGCATGCACAGGGCCGC |
| *Darpp32* | CCCGACAGGTGGAGATGATC | GCTGCACAGCTTTCAGTGATG | CTGCCATGCTTTTCCGGGTCTCAGA |
| *Drd2* | ACACCACTCAAGGGCAACTGT | GGCGGGCAGCATCCA | CCCTGAGGACATGAAACTCTGCACCG |
| *Hdac7* | CCCACCTGTCAGACCCAAGT | AGTCATAGACCAGCCCTGTAGCA | CTCAACAGCTCAGAGACA |
| *Htt* | GCTGCACCGACCGTGAGT | CGCAGGCTGCAGGGTTAC | CAGCTCCCTGTCCCGGCGG |
| *Igfbp5* | AAGGATTCTACAAGAGAAAGCAGTGTAA | ACTTGTCCACACACCAGCAGAT | TCCCGTGGCCGCAAACGTG |
| *Pcp4* | CTGAGCTGTTCTGTGGGACCTA | CGCTCCGGCACTTTGTCT | CTGCGGAGTCAGGCCAACATGA |
| *Penk1* | ATGCAGCTACCGCCTGGTT | GCAGCTGTCCTTCACATTCCA | AGGCGACATCAATTTCCTGGCGTG |
| *Psme1* | TGATGACCAACCTTCACACCAA | TCACCCCTCTCGGAGAAGTACT' | CTGGAAGGCTTCCACACGCAGATCTCC |
| *UchL1* | GGTACCATCGGGTTGATCCA | AACTGTTTCAGGACGGATCCA | AACCAAGACAAGCTGGAATTTGAGGA |

**KEY: Cnr1** (Cannabinoid receptor 1); **Darpp32** (Dopamine and cAMP regulated neuronal phosphoprotein, also known as Ppp1r1b, protein phosphatase 1, regulatory subunit 1B); **Drd2** (Dopamine D2 receptor); **Hdac** (histone deacetylase); **Htt** (Human Huntington’s disease gene, used to detect human exon 1 transgene); **Igfbp5** (insulin-like growth factor binding protein 5 precursor); **Pcp4** (Purkinje cell protein 4); **Penk1** (Preproenkephalin); **Uchl1** (ubiquitin carboxyl-terminal hydrolase L1).
